# Practical Notes and Formulæ

**Published:** 1888-07

**Authors:** 


### PRACTICAL NOTES AND FORMULÆ.

**For External Piles and Pruritus Ani.**—

℞. Bismuth oleate.................................ʒ ij,

Extract opii.................................gr. x,

Extract. belladonna.................................gr. x,

Cerati simplecis.................................℥ ss,

Cocaine.................................gr. xxx.

M. Apply as required.

**Mistura Acidi Muriatici.**—

℞. Acidi hydrochlorici.................................*m* xxxv,

Tinct. gentianæ comp.................................fl ℥ ii,

Aquæ.................................fl ℥ ij,

M. Dose—a teaspoonful in half glass of water as a tonic.—*Medical World.*

**Mistura Anti-Emetica.**—

℞. Creasoti.................................*m* xij,
Acidi hydrocyanici dilute.................................*m* xxx,
Acaciæ pulv.................................
Sacchari pulv.................................aa ʒ vi,
Aquæ.................................q. s. ad. fl. ℥ iv,

M. Dose—a teaspoonful.—*Medical World.*

**Mistura Rhei et Calcis.**—

℞. Tinct. opii camphor.................................
Syr. rhei aromat.................................aa fl ʒ ij,
Liquoris calcis.................................℥ ij.

M. Dose—a teaspoonful for children in diarrhœa.—*Ib.*

**Compound Mixture of Muriate of Ammonia.**—

℞. Ammonia mur.................................gr. clx,
Syr. senege.................................f. ℥ ij,
Morph. mur.................................gr. j,
Syr. scillæ.................................f. ℥ viij,
Syr. pruni virg.................................f. ℥ vi,
Ol. gaultheria.................................gtt. lxxx.

Make a mixture. In diseases of lungs, bronchi, etc.
Dose—one teaspoonful, according to indications.—*Med. World.*

**Pile Ointment.**—

℞. Ext. belladon.................................℈ ij,

Iodoformi.................................

Plumbi acetatis.................................aa gr. viij,

Petroleum jelly.................................℥ j.

M. ft. ung. S. Apply three or four times a day.—*Ib.*

**Antipyrin in Gonorrhoæa.**—The following solution is another proof of the extent to which antipyrin is invading medical practice. It has had great success in gonorrhœa, both in the acute and chronic forms:

℞. Dist. rose water........................

Dist. cherry laurel water...............aa grms. c,

Sulphate of zinc.........................centigrams l,

Antipyrine.................... .........grammes v.

M. S —Inject two or three times a day, as usual.—*Thomas Linn, M. D., in Medical Times.*

**Sore Nipples.**—

℞. Boracic acid..................................gr. xx,
Mucilage of gum arabic........................℥ j.

M. Dry the nipple with a soft cloth or a bit of absorbent cotton, and apply with a camel's hair brush after each time the child is removed from the breast.—*Medical and Surgical Synopsis.*

**Chronic Bronchitis.**—

℞. Tinct. capsici..................................ʒ ss,

Tinct. nucis vomicæ............................ʒ ij,

Tinct. sanguinariæ.............................ʒ i,

Vini pici........................................℥ iv.

M. Sig.—Dessertspoonful four times a day.—*Ib.*

**Hair Tonic.**—The following is reported as an excellent hair tonic:

℞. Acid carbolic..................................ʒ ss,

Tr. nux vom.....................................ʒ ii,

Tr. cinchona....................................℥ j,

Tr. cantharides................................ ʒ ss,

Aq. cologne................................

Ol. cocoa......... .................aa. q. s. ad ℥ iv,

M. Sig.—Apply once a day to the scalp by means of a soft sponge· This will prevent the hair from falling out, if it does not produce a luxuriant crop. The head should be bathed in pure water every morning, then dressed.—*Med. and Surgical Synopsis.*

**Hemorrhoids.**—

℞. Ergotine.................................. ....fl. ʒ ij,

Sulphate of morphine.........................gr. vi,

Lanolin.................................troy ℥ j.

M. Sig.—To be used locally.—*Ib.*

**Hydrochloric Acid and Cocaine** may be given as follows:

℞. Syrup. menth. piper............................℥ vii¾.

Acid. hydrochloric...........................*m* xv.

Cocain. hydrochlorat..........................gr. jss.

Which is prescribed by Huchard in varying doses for gastric pain in eating.

